# Detection of Oropouche and Punta Toro Virus Infections by Enhanced Surveillance, Panama, 2023–2024

**DOI:** 10.3201/eid3201.251224

**Published:** 2026-01

**Authors:** Maria Chen-Germán, Claudia González, Dimelza Araúz, Celestino Aguilar, Melanie Vega, Oris Chavarria, Ambar Moreno, Erika Santiago, Danilo Franco, Elimelec Valdespino, Jessica Gondola, Mayla Pinedo, Domicio Espino, Tamara Salcedo, Leticia Franco, Nicanor Obaldía, Blas Armien, Alexander A. Martínez, Brechla Moreno

**Affiliations:** Instituto Conmemorativo Gorgas de Estudios de la Salud, Panama City, Panama (M. Chen-Germán, C. González, D.J. Araúz, M. Vega, O. Chavarria, A. Moreno, E. Santiago, D. Franco, E. Valdespino, J. Gondola, N. Obaldia III, B. Armien, A.A. Martínez, B. Moreno); University of Panama, Panama City (C. Gonzalez, C. Aguilar, A.A. Martínez); Dr. Hugo Spadafora Polyclinic, Colon City, Panama (M. Pinedo); Ministry of Health, Penonome City, Panama (D. Espino); Ministry of Health, Panama City (T. Salcedo, B. Armien); Organización Panamericana de la Salud/Organización Mundial de la Salud, Panama City (L. Franco); Sistema Nacional de Investigación-SENACYT, Panama City (B. Armien, A.A. Martínez); Interamerican University of Panama, Panama City (B. Moreno)

**Keywords:** viruses, vector-borne infections, surveillance, Oropouche virus, Punta Toro virus, arboviruses, Panama

## Abstract

Enhanced arboviral surveillance in Panama revealed an Oropouche virus case, 5 months before the 2025 national outbreak, in samples that tested negative for routinely screened arboviruses. Subsequent contact tracing identified an additional case of Punta Toro virus. Our findings highlight the importance of expanding diagnostic efforts to identify circulating arboviruses.

In recent years, arbovirus incidence and geographic range have expanded markedly, leading to multiple outbreaks worldwide. Nevertheless, a substantial proportion of patients remain undiagnosed, largely because of the predominant focus on dengue clinical suspicion. We report findings from an enhanced surveillance initiative in Panama that reanalyzed dengue-negative samples, leading to the identification of other viral agents, including Oropouche virus (OROV) and Punta Toro virus (PTV). Our results highlight the need to broaden diagnostic approaches in arbovirus endemic regions.

OROV is a single-stranded, negative-sense, segmented RNA virus classified within the family *Peribunyaviridae*, genus *Orthobunyavirus*, primarily transmitted by the anthropophilic biting midge, *Culicoides paraensis*. OROV, first identified in Trinidad and Tobago in 1955, has circulated across Central and South America (*1*). In Panama, it was first isolated in 1989, although there is serologic evidence of earlier circulation in 1968 and 1978 (*2*).

In 2024, the Pan American Health Organization/World Health Organization reported rising OROV cases across the Americas, including areas with no previous evidence of circulation (*3*). Health officials linked this geographic expansion to severe clinical outcomes, including fatalities, vertical transmission, fetal loss, and microcephaly (*4*). Studies from Brazil identified a novel recombination event in OROV genomes (*5*).

PTV belongs to the *Phenuiviridae* family, *Phlebovirus* genus. This highly diverse genus encompasses numerous species globally distributed and transmitted by vectors like sandflies and ticks (*6*). Key members include Toscana virus and Rift Valley fever virus, which cause epidemics and neurologic disease across the Mediterranean Basin, Europe, and the Middle East (*7*). Reports from the Americas have identified *Phlebovirus* species in Brazil, Peru (*8*), Colombia (*9*) and Panama (*10*), although PTV appears to be reported only in Panama. Human infections, mainly detected in metropolitan areas of Panama, cause nonspecific febrile illness characterized by fever, headache, weakness, and retro-orbital pain (*10*).

## The Study

In 2023–2024, the Gorgas Memorial Institute (Panama City, Panama) analyzed 3,589 serum samples. After a Pan American Health Organization/World Health Organization alert on rising regional OROV incidence and because of the lack of confirmed cases in Panama, we retrospectively tested a subset (24%) of dengue virus (DENV)–negative samples from that set for OROV by reverse transcription PCR (*11*). We later integrated this assay into a broader diagnostic algorithm for samples testing negative for DENV, Zika virus, and Chikungunya virus, which included testing for *Alphavirus*, *Orthoflavivirus*, and *Phlebovirus* (Appendix 1).

Through this surveillance, we identified an OROV-positive case from August 2024. The patient, a 31-year-old male environmental officer stationed in a forested area of the Soberanía National Park, Panama, sought treatment for an acute febrile syndrome characterized by severe frontal-occipital headache, chills, arthralgia, and marked asthenia. The man underwent an initial clinical evaluation at a primary healthcare facility, where the result of a dengue NS1 antigen test was negative. Treating clinicians reported no neurologic manifestations or relapses. Health officials conducted contact tracing, covering the patient’s residence and workplace, collecting serum and urine samples from 36 contacts. Twelve symptomatic persons underwent testing for OROV, DENV, Zika virus, chikungunya virus, *Orthoflavivirus*, *Phlebovirus*, and *Alphavirus* (Table 1). Of those, 1 contact tested positive for the *Phlebovirus* genus, with symptoms including fever, chills, myalgia, arthralgia, headache, retroorbital pain, and conjunctivitis.

**Table 1:** Signs and symptoms found in symptomatic contacts of case-patient with Oropouche virus from study of detection of Oropouche and Punta Toro virus infections by enhanced surveillance, Panama, 2023–2024

Sign/symptom	No. (%) contacts, n = 12
Fever >38°C	8 (67)
Severe headache	8 (67)
Intense chills	7 (58)
Retro-orbital pain	4 (33)
Conjunctivitis	2 (17)
Body pain	1 (8)

We sequenced aliquots from both positive samples (OROV and *Phlebovirus* genus) using an in-house metagenomic approach (A. Martinez et al., unpub. data, https://dx.doi.org/10.17504/protocols.io.36wgq6545lk5/v1) (Appendix 1), yielding a complete OROV genome (GenBank accession nos. PV942050–2) (Table 2). We performed phylogenetic analysis using the Nextstrain OROV database (https://github.com/nextstrain/oropouche), which placed the sample at the basal node of the BR-2015–2024 clade (*5*), notably separated from the sequences circulating in the Brazil outbreak (Figure).

**Table 2:** Sequence analysis results from a study of detection of Oropouche and Punta Toro virus infections by enhanced surveillance, Panama, 2023–2024*

Organism and segment	GenBank accession no.	Start position	End position	No. reads	Base coverage	Coverage, %	Mean depth of segment, reads, n	Mean base quality, %	Mean mapped read quality, %
Oropouche virus sample A003066									
M	PP154171	1	4371	6,387	4,364	99.4	162.4	37.5	41.3
S	PP154170	1	944	3,411	846	89.6	405.13	37	45.5
L	PP154172	1	6814	14,128	6,814	100	239.7	37.5	46.2
Punta Toro virus sample A3416V2									
S	KP272018	1	1899	217,265	1,899	100	18.609	37	48.2
M	KP272017	1	4340	280,677	4,340	100	10.978	37	50.3
L	KP272016	1	6407	877,321	6,407	100	23.679	37	51.2

*L, large; M, medium; S, small.

**Figure:**
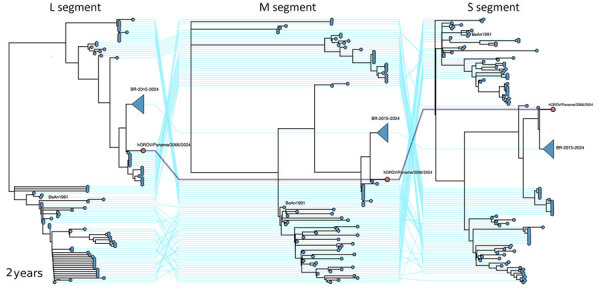
Concatenated maximum likelihood phylogenetic tree of 3 OROV segments from a patient found in study of detection of OROV and Punta Toro virus infections by enhanced surveillance, Panama, 2023–2024. The Panama sample is labeled as hOROV/Panama/3066/2024. Also shown in the tree are the prototype reference strain BeAn19991 and the Brazil outbreak clade BR-2015-2024, collapsed for better visualization. L, large; m, medium; OROV, Oropouche virus; s, small.

We performed a comparative analysis of OROV using the Panama sequence (hOROV/Panama/A003066/2024), recent Brazil sequences (OROV/Saul/17225/2020, LACENAM_ILMD_3228ZCF, and ILMD_TF29; GenBank accession nos. PP154170–2), and the ancestral BeAn19991 strain as reference. The Panama sequence showed multiple amino acid substitutions across structural and nonstructural proteins sharing 96.8% amino acid identity for the large (L) segment, 97%–99% for the medium (M) segment, and 98%–100% for the small (S) segment with BeAn19991. The Panama sequence also shared 99% amino acid identity for the L segment, 98%–99% for the M segment, and 100% for the S segment with the Brazil sequences (Appendix 1 Figure 1). We theorized that the differences may influence viral replication or transcription efficiency (K.B. Gunter et al., unpub. data, https://dx.doi.org/10.1101/2025.08.02.668287).

Further testing identified the *Phlebovirus* genus–positive sample as PTV (GenBank accession nos. PV942053–5) (Appendix 1 Figure 2; Appendix 2). Compared against the prototype strain (GenBank accession nos. KP272028–30), amino acid identity was 87% for the L segment, 94% for the M segment, and 95%–97% for the S segment. Comparison with a 2004 sequence (GenBank accession nos. KP272031–3) showed amino acid identity as 99% with the L segment, 99% with the M segment, and 98%–99% with the S segment (Appendix 1 Figures 3, 4).

Before the OROV outbreak that began in January 2025 in Panama, the last documented case occurred in 1989 in Bejuco, a coastal community ≈35 miles west of Panama City, a region surrounded predominantly by mature broadleaf forest at the time (*2*). The OROV case we identified was likely acquired in a similar ecologic setting, at the interface between the metropolitan region and the Soberanía National Park. The geographic proximity between Bejuco and Soberanía National Park (22–25 miles) suggests shared ecologic characteristics and supports the hypothesis of ongoing cryptic OROV circulation before the 2025 cases reported in Darién province.

Although the absence of genomic data over the intervening decades limits reconstruction of OROV’s full evolutionary history in Panama, our case provides valuable evidence suggestive of persistent, undetected sylvatic circulation. The Panama OROV sequence shows marked amino acid divergence from the ancestral BeAn19991 strain, especially in the L and M segments, and fewer differences relative to strains currently circulating in Brazil and South America, which have been reported since 2022 (*5*). Those changes are similar to mutations reported in recent studies (G.C. Scachetti et al., unpub. data, https://dx.doi.org/10.1101/2024.07.27.24310296). The differences between the Panama strain and newly described OROV reassortants suggests ongoing adaptive processes, driven potentially by local selective pressures or prolonged cryptic circulation in Panama. Sporadic retrospective OROV detections in Brazil from dengue surveillance samples mirror this context (*12*).

PTV, causing undifferentiated febrile illness (*10*), is an arbovirus that appears to be underreported in Panama, which complicates clinical suspicion required for an accurate diagnosis. Our sequenced sample revealed appreciable genetic variability compared with the reference strain but fewer differences relative to a more recent sequence, suggesting both divergence from the original reference and ongoing viral evolution.

## Conclusions

Both of the case-patients we describe had worked in areas of mature broadleaf forest, suggesting those locations as the sites of infection and indicating possible simultaneous, cryptic circulation of different arboviruses. The enhanced arboviral surveillance described in our study, which involved testing across multiple viral genera, broadened the analysis of dengue-negative samples, enabling the detection of other arboviruses. Of note, the OROV and PTV strains in our study showed greater genetic similarity among recent strains, pointing to ongoing evolution, while divergence from older strains reflects accumulation of mutations over time. Together, those findings underscore the complexity and dynamic nature of arbovirus evolution and highlight the crucial need to enhance and expand arboviral surveillance frameworks beyond the routine detection of dengue to encompass the full spectrum of circulating arboviruses and their potential effect on public health.

Appendix 1 Additional information for detection of Oropouche and Punta Toro virus infections by enhanced surveillance, Panama, 2023–2024.

Appendix 2 Metadata related to detection of Oropouche and Punta Toro virus infections by enhanced surveillance, Panama, 2023–2024
