# Supplementary material for: Detection of Oropouche and Punta Toro Virus Infections by Enhanced Surveillance, Panama, 2023–2024
Source: Emerg Infect Dis. 2026 Jan;32(1):114–7. doi: 10.3201/eid3201.251224 (PMC12870164; doi:10.3201/eid3201.251224)
Supplement: Appendix 1 — Additional information for detection of Oropouche and Punta Toro virus infections by enhanced surveillance, Panama, 2023–2024. [file 25-1224-Techapp-s1.pdf]

Article DOI: http://doi.org/10.3201/eid3201.251224

EID cannot ensure accessibility for supplementary materials supplied by authors. Readers who have difficulty accessing supplementary content should contact the authors for assistance.

Detection of Oropouche and Punta Toro Virus Infections by Enhanced Surveillance, Panama, 2023–2024

Methods

Extraction, Pool Assembly, and Generic Protocols

Negative samples tested for DENV, ZIKV, and CHIKV were extracted using MagMAX Viral/Pathogen II Nucleic Acid Isolation Kit (Thermo Fisher Scientific, Cat.A48383) in the KingFisher Flex according to the manufacturer's instructions. With the RNA, pools were prepared by combining five samples per pool, with 8 µL from each sample. Pools were then tested using generic protocols for different viral genera:

- *Orthoflavivirus:* This generic protocol detects West Nile virus (WNV), St. Louis encephalitis (SLE), yellow fever virus (YFV), and all four dengue serotypes (DENV1–4). Additionally, in practice, we have also detected Zika virus (ZIKV), not shown in the original paper (*1*).
- *Alphavirus:* This protocol detects seven virus species Western Equine Encephalitis (WEE), Eastern Equine Encephalitis (EEE), Venezuelan Equine Encephalitis (VEE), Sindbis (SIN), Ross River (RR), Chikungunya (CHIK), and Semliki Forest (SF). Furthermore, Madariaga virus (MADV) has also been detected in practice, although it was not included in the original paper (*2*).
- *Phlebovirus* : This protocol detects a wide variety of species of the Phlebovirus genus, including: Alenquer virus, Chandiru virus, Chagres virus, Punta Toro virus, Rift Valley fever virus, Naples sandfly fever virus, Toscana virus, Sicilian sandfly fever virus, Cacao virus, Buenaventura virus, Frijoles virus, Rio Grande virus, among others (*3*).

All of them were amplified with AgPath-ID One-Step RT-PCR Reagents (Thermo Fisher Scientific, Cat.4387424. Amplified products were revealed using a 2% agarose gel in TBE.

OROV and PTV consensus generation

Obtained reads were filtered and index trimmed with fastp v0.24.0 using default parameters, then kraken2 (*4*) was used to classify human genome reads. Unclassified reads were used for mapping against OROV references for S, M, and L genome segments (PP154170–72), with minimap v4.0.0 (*5*), mapped reads were sorted with samtools v1.14 (*6*), uncovered regions on the reference genomes were masked using bedtools v2.30.0, and a consensus call was done using bcftools v1.14.

Phylogenetic analysis of Oropouche virus (OROV):

Segments generated were visually inspected using Geneious Prime v2025.0.3 (*7*). An available OROV genome database was used to evaluate the obtained genome's phylogenetic relationships (https://nextstrain.org/oropouche) (*8*). The phylogeny analysis was built using a Nextstrain repository, and the anglegram was prepared using Baltic (https://github.com/evogytis/baltic).

Phylogenetic analysis of Punta Toro virus (PTV):

Segments generated were visually inspected using Geneious Prime v2025.0.3. Subsequently, the obtained sequence for Segment L was aligned against reference sequences from virus arthropod-borne described in phylogenetic metadata (Table 2). Using the multiple alignment tool, MAFFT, version 7.490 (*9*), the aligned FASTA file was used for the construction of the phylogenetic tree TPM2u+F+G4 using the IQ-TREE multicore version 2.2.0 software. The FigTree version 1.4.4 (*10*) application was used to visualize the phylogenetic tree.

OROV Genomic Mutation Analysis

A multiple sequence alignment of proteins from one Panamanian OROV strain was performed using ILMD_TF29_Brazil_AM_Tefe as a reference. subsets of protein sequences

were analyzed. The Snipit pipeline (https://github.com/aineniamh/snipit) (*11*) was used to identify amino acid mutations among four strains.

References

1. Ayers M, Adachi D, Johnson G, Andonova M, Drebot M, Tellier R. A single tube RT-PCR assay for the detection of mosquito-borne flaviviruses. J Virol Methods. 2006;135:235–9. PubMed

2. Sánchez-Seco MP, Rosario D, Quiroz E, Guzmán G, Tenorio A. A generic nested-RT-PCR followed by sequencing for detection and identification of members of the alphavirus genus. J Virol Methods. 2001;95:153–61. PubMed

3. Sánchez-Seco MP, Echevarría JM, Hernández L, Estévez D, Navarro-Marí JM, Tenorio A. Detection and identification of Toscana and other phleboviruses by RT-nested-PCR assays with degenerated primers. J Med Virol. 2003;71:140–9. PubMed

4. Wood DE, Lu J, Langmead B. Improved metagenomic analysis with Kraken 2. Genome Biol. 2019;20:257. PubMed

5. Li H. Minimap2: pairwise alignment for nucleotide sequences. Bioinformatics. 2018;34:3094–100. PubMed

6. Li H, Handsaker B, Wysoker A, Fennell T, Ruan J, Homer N, et al.; 1000 Genome Project Data Processing Subgroup. The Sequence Alignment/Map format and SAMtools. Bioinformatics. 2009;25:2078–9. PubMed

7. Drummond AJ, Ashton B, Buxton S, Cheung M, Cooper A, Duran C, et al. Geneious. Biomatters Ltd. 2011

8. Hadfield J, Megill C, Bell SM, Huddleston J, Potter B, Callender C, et al. Nextstrain: real-time tracking of pathogen evolution. Bioinformatics. 2018;34:4121–3. PubMed

9. Katoh K, Rozewicki J, Yamada KD. MAFFT online service: multiple sequence alignment, interactive sequence choice and visualization. Brief Bioinform. 2019;20:1160–6. PubMed

10. Rambaut A, Drummond A. FigTree: Tree figure drawing tool, v1. 4.2. Institute of Evolutionary Biology, University of Edinburgh. 2012

11. O’Toole Á, Aziz A, Maloney D. Publication-ready single nucleotide polymorphism visualization with snipit. Bioinformatics. 2024;40:btae510. PubMed

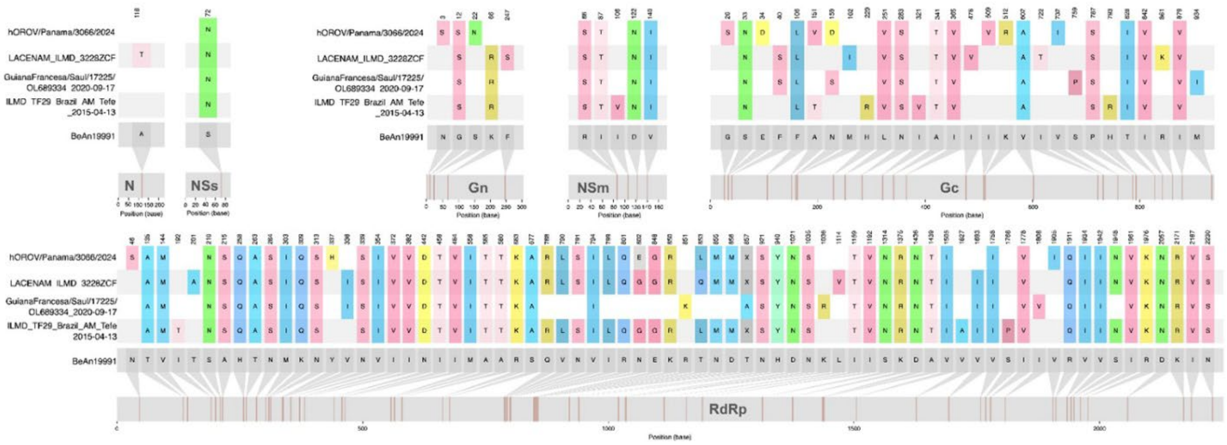

Appendix 1 Figure 1. A study of enhanced surveillance revealing cases of Oropouche and Punta Toro viruses, Panama, 2023–2024 included a comparison of the Panamanian sequence with BeAn19991, revealing 61 substitutions in segment L (RdRp), 25 in segment M (Gn, NSm, Gc), and one in segment S (NSs). Relative to the Brazilian outbreak sequence LACENAM_ILMD_3228ZCF, we observed 8 substitutions in segment L (RdRp), 16 in segment M (Gn, NSm, Gc), and 1 in segment S (N). We rendered this figure using the Snipit tool, which utilizes the pipeline for amino acid visualization (https://github.com/aineniamh/snipit).

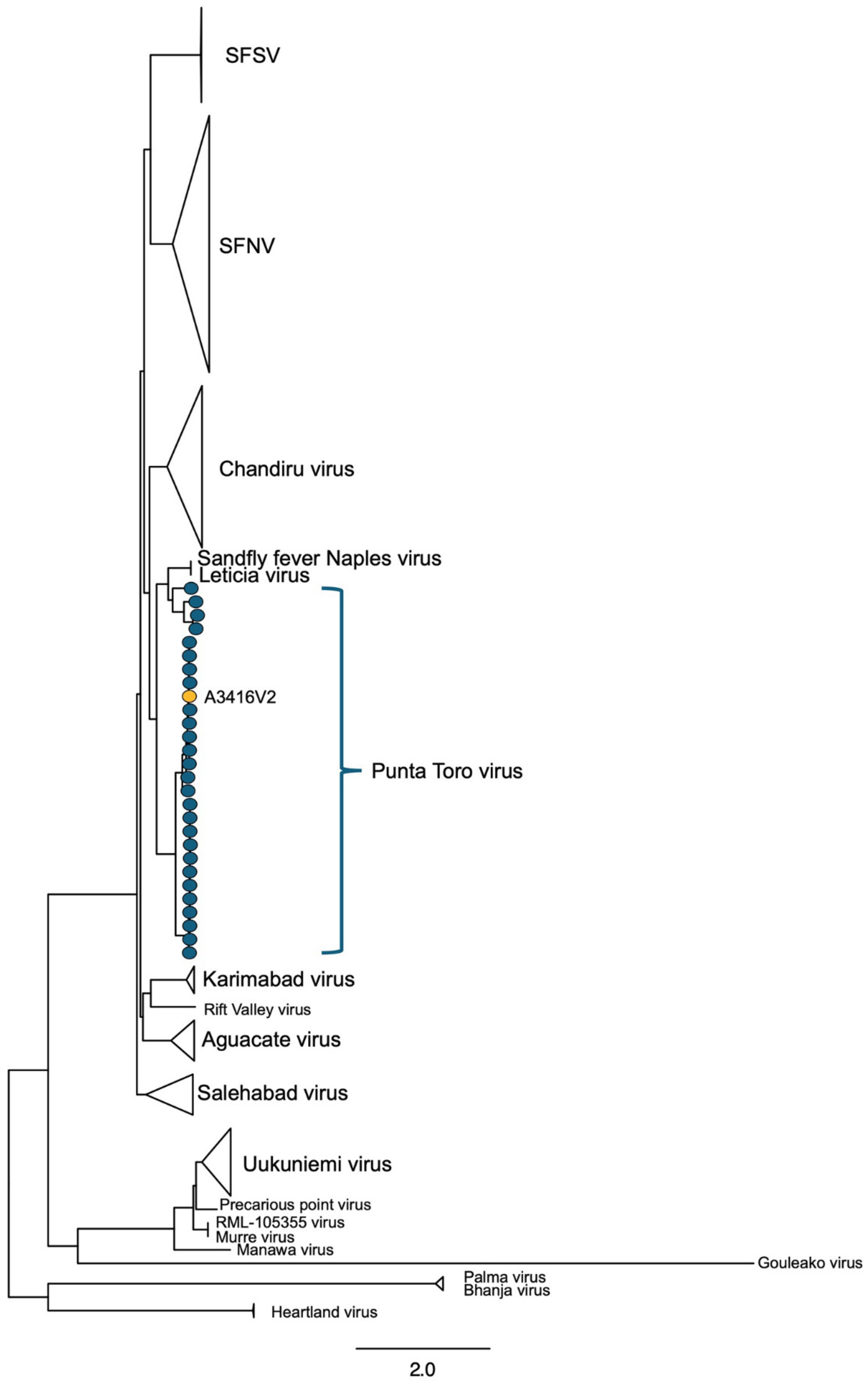

Appendix 1 Figure 2. Maximum likelihood phylogenetic tree constructed with IQ-TREE software (https://iqtree.github.io) under TPM2u+F+G4 model. Dataset comprises viruses described in Appendix 2 (https://wwwnc.cdc.gov/EID/article/32/1/25-1224-App1.xlsx). Sample A3416V2 in yellow cluster with reference sequences from Punta Toro virus.

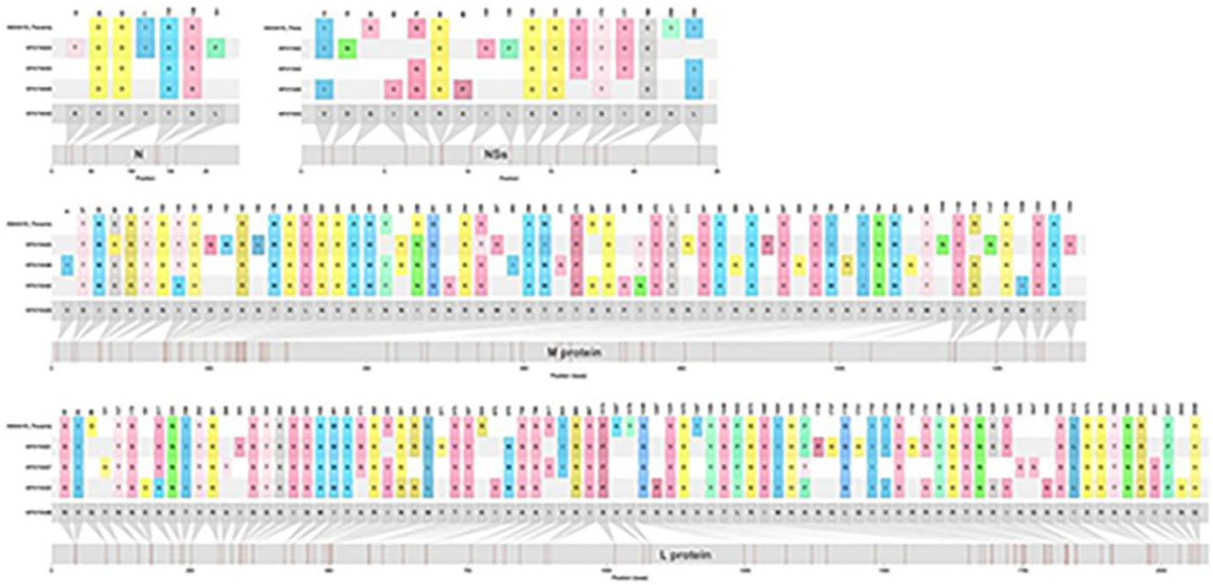

Appendix 1 Figure 3. Amino acid comparison between the Panamanian sequence and the Punta Toro virus prototype reference strain (KP272028–KP272030) shows differences in segment S for protein N (5 substitutions) and NS (12 substitutions), in segment M (43 substitutions), and in segment L (68 substitutions).

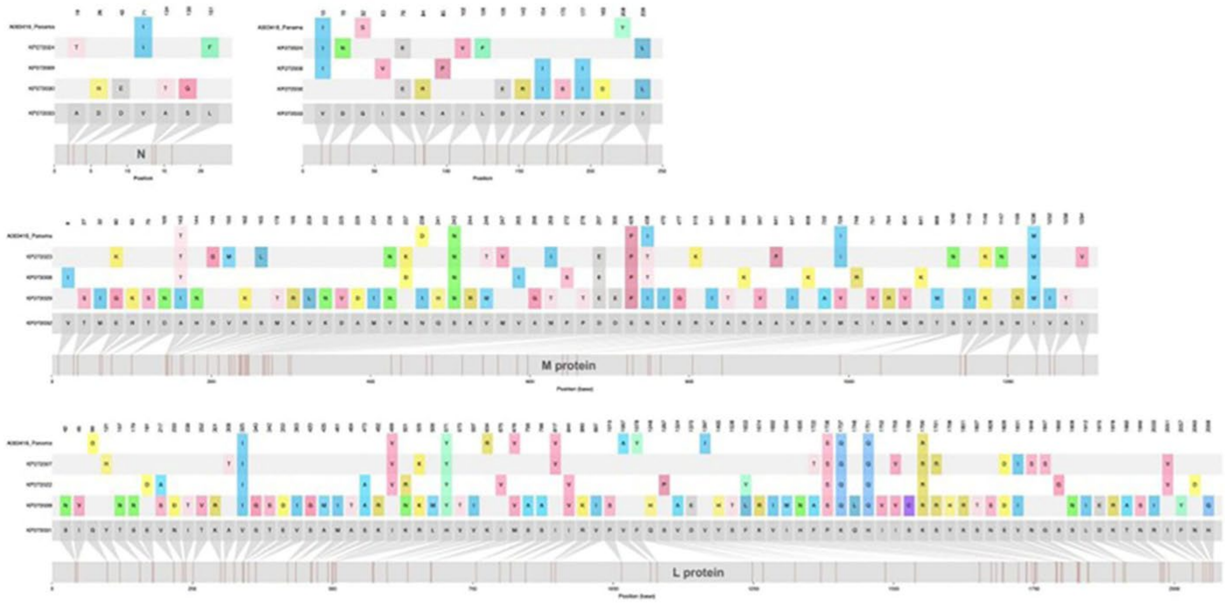

Appendix 1 Figure 4. Amino acid comparison between the Panamanian sequence and the Punta Toro virus strain GML 488831 year 2004; (KP272031–KP272033) shows differences in segment S for protein N (1 substitution) and NS (3 substitutions), in segment M (7 substitutions), and in segment L (14 substitutions).
